# Awareness about Breast Cancer and Breast Self-Examination among Female Students at the University of Sharjah: A Cross-Sectional Study

**DOI:** 10.31557/APJCP.2019.20.6.1901

**Published:** 2019

**Authors:** Syed Azizur Rahman, Amina Al–Marzouki, Michael Otim, Nour El Hoda Khalil Khayat, Reham Yousef, Prama Rahman

**Affiliations:** 1 *College of Health Sciences, University of Sharjah, United Arab Emirates, *; 2 *Simon Fraser University, Canada. *

**Keywords:** Breast cancer, awareness, breast cancer screening, breast self-examination, students, United Arab Emirates

## Abstract

**Background:**

Breast cancer is a leading cause of mortality among women in the United Arab Emirates (UAE). Many young women in the UAE have poor knowledge about breast cancer screening, including risk factors and warning signs/symptoms. We investigated awareness about breast cancer and breast self-examination (BSE) as a screening tool among female students at the University of Sharjah, UAE.

**Methods:**

This study used a cross sectional survey design. Participants were 241 undergraduate female students (aged ≥18 years) from three University of Sharjah campuses. Data were collected from March to April 2017 using a self-administered questionnaire. The questionnaire covered: sociodemographic characteristics; knowledge about breast cancer, risk factors, and warning signs/symptoms; and knowledge and practice of BSE. Data were analyzed using descriptive statistics and Pearson’s chi-square tests.

**Results:**

About 38.6% of participants were from the Medical campus, 37.3% were from the Women’s campus, and 24% were from the Fine Arts and Design campus. Most (99%) participants had heard of breast cancer. About 50% were knowledgeable about the risk factors, but only 38% were knowledgeable about warning signs/symptoms. The most commonly identified risk factors were family and personal histories of breast cancer, and the most commonly identified warning sign/symptom was breast lump. There was a significant association between knowledge about risk factors and campus type. Participants from the Medical campus were more knowledgeable about risk factors than participants from the other two campuses. Overall, 68.5% of participants had heard of BSE, but few participants actually performed BSE. Reasons for not performing BSE included “forgetting” and “not knowing how.”

**Conclusions:**

Although most participants were aware of breast cancer, knowledge about risk factors and warning signs/symptoms was relatively poor. Knowledge about performing BSE was particularly low. This highlights the importance of increasing awareness about breast cancer and BSE among young women in the UAE.

## Introduction

Breast cancer claims the lives of hundreds of thousands of women each year and affects countries at all levels of modernization (National Breast Cancer Foundation, 2016). In 2018, there were 2.1 million newly diagnosed breast cancer cases among women, accounting for almost one in four cancer cases among women (Bray et al., 2018). The World Health Organization (WHO) reported that an estimated 627,000 women died from breast cancer in 2018, representing approximately 15% of all cancer deaths among women (WHO, 2019). Breast cancer is the second most common cancer in the world and the most frequent cancer among women, with an estimated 1.67 million new breast cancer cases diagnosed in 2012 (Farley et al., 2015; Noreen et al., 2015). Globally, breast cancer represents about 12% of all new cancer cases, and 25% of all cancer cases in women (World Cancer Research Fund, 2015). Despite the high incidence rate, around 89% of women in Western countries diagnosed with breast cancer are still alive 5 years after diagnosis, with this high survival rate attributed to early detection and treatment (Worldwide Breast Cancer, 2011). Cancer that is diagnosed at an early stage when it is not too large and has not yet spread is more likely to be treated successfully (Cancer Research UK, 2018). It is estimated that one-third of all cancers can be prevented, and a further third of all cancers may be cured if diagnosed at an early stage (Noreen et al., 2015).

Breast cancer is also the most common cancer type in the United Arab Emirates (UAE), accounting for almost 60% of all cancer cases seen at Dubai Hospital (Emirates 24/7 News, 2016). In the Middle East and Gulf region, the incidence of breast cancer is rising and affecting a younger population compared with Western countries (Radi, 2013). According to the WHO, the incidence of cancer in the Middle East region (including the UAE) is increasing, with the number of cases expected to double by 2030 (Chaudhary, 2018; Rizvi, 2016). The Health Department of Abu Dhabi reported that in the UAE alone, there are 4,500 new cases of cancer each year, with breast, colorectal, and lung cancers being most common (Chaudhary, 2018). Reports from medical institutions under the UAE Ministry of Health indicated that 39 women died of breast cancer in the UAE in 2013 (Emirates 24/7 News, 2014). This is supported by Health Authority of Abu Dhabi statistics that show breast cancer is the leading type of cancer causing death among women in the UAE (Health Authority of Abu Dhabi, 2014). However, the average age at diagnosis of breast cancer among UAE women is about 10 years younger than women in Europe and the United States (45–55 years vs. 55–65 years) (Emirates 24/7 News, 2014). Given these statistics, it is particularly concerning that a study conducted in Ajman, UAE, reported a high proportion of female university students had inadequate knowledge about breast cancer (Al-Sharbatti et al., 2014). This suggests that raising breast cancer awareness could contribute to reducing the number of breast cancer cases among young women in the UAE. 

Breast self-examination (BSE), clinical breast examination, and mammography are commonly recommended screening methods (Noreen et al., 2015). BSE is a screening technique for early breast cancer detection that can be performed by women at home. This is a simple, inexpensive, easy, and effective technique that allows women to examine their breast tissue for any physical or visual changes. BSE increases women’s chances for treatment, thereby increasing the survival rate in women (Erbil and Bolukbas, 2014). BSE can help screen for tumors, cysts, and other abnormalities in the breasts. The American Cancer Society recommends BSE for early detection of breast cancer as it assists women to become familiar with the appearance and sense of their breasts, and helps them to detect any changes in their breasts as soon as possible (American Cancer Society, 2008). In resource constrained settings such as Nigeria, BSE has been reported to be culturally and religiously acceptable, friendly, and incurring no cost (Oladimeji et al., 2015). If detected early, breast cancer can be treated in the early stages of the disease, meaning BSE is something all women should prioritize. Despite advances in treatment, detecting breast cancer as early as possible is important to maximize the potential for good health outcomes. Organizations concerned with breast health education suggest that all women should start performing BSE regularly as soon as their breasts are fully developed. For example, the Maurer Foundation suggests BSE should be performed at least once a month from age 18 years (Maurer Foundation, 2018). Such regular examination means women become familiar with their own breasts and are therefore more likely to detect any changes.

Awareness about breast cancer is an important factor that has a major impact on the incidence and outcomes of the disease (Noreen et al., 2015). For example, if female university students have sufficient knowledge about breast cancer, they can help prevent cancer in themselves and contribute to reducing the incidence of breast cancer in their community. To date, knowledge about breast cancer and practice of BSE has not been assessed among the female student population of the University of Sharjah, UAE. 

Therefore, we aimed to assess the awareness of breast cancer and BSE among female students at the University of Sharjah. In particular, the objectives of this study were to:

1) Assess knowledge regarding risk factors and warning signs and symptoms of breast cancer and the practice of BSE among female students at the University of Sharjah; and 

2) Assess the association between campus type (Medical, Women’s, and Fine Arts and Design) and knowledge of risk factors and warning signs/symptoms of breast cancer.

## Materials and Methods

*Study design, setting, and population*

We used a cross sectional survey to explore the level of awareness about breast cancer and BSE among female students at three University of Sharjah campuses (Medical, Women’s, and Fine Arts and Design) in the UAE. The target population was undergraduate female students aged ≥18 years from the three campuses. 

We excluded students who were male, female students younger than 18 years, students attending other campuses, and female students who were non-English speakers. The sample size was calculated as 300 female students following a purposive sampling method. However, because of limited time and resources, 250 questionnaires were distributed to female students who agreed to participate in this study. In total, 241 responses were received. 

*Data collection*

A questionnaire was developed for this study based on questionnaires used in similar previous studies conducted in Egypt (Boulos and Ghali, 2014), Nigeria (Makanjuola et al., 2013), North-East London (Forbes et al., 2010), and Turkey (Karayurt et al., 2008). The questionnaire comprised three sections: participants’ sociodemographic characteristics (Section 1); knowledge about breast cancer, risk factors, and early warning signs and symptoms (Section 2); and knowledge about and practice of BSE (Section 3). Data collection took place between March 2017 and April 2017.

Demographic information collected included age, campus (Medical, Women’s, Fine arts and Design), academic year, and marital status. Fourteen questions were used to investigate participants’ knowledge of risk factors and signs/symptoms. Participants who correctly answered seven or more of these 14 questions were considered knowledgeable; those who correctly answered six or fewer questions were considered non-knowledgeable. In addition, the questionnaire included 10 questions about the warning signs and symptoms of breast cancer. Participants who correctly identified five or more warning signs/symptoms were considered knowledgeable, and those who correctly identified four or fewer were considered non-knowledgeable. 

The final part of the questionnaire assessed participants’ knowledge about the purpose of BSE (e.g., “BSE is to detect lumps in the breast”), how often BSE should be performed (e.g., “weekly,” “monthly”), and when BSE should be performed (e.g., “before menstruation,” “any time”). In addition, this section evaluated whether participants performed BSE, reasons for not performing BSE, how often BSE was performed (for those that performed BSE), and if participants were confident about detecting changes in their breasts. 

*Data analysis*

After data collection, data were organized, coded, and tabulated using SPSS version 22 (IBM Corp. Armonk, NY) and Microsoft Office Excel. The statistical analyses included descriptive statistics and Pearson’s chi-square tests.

*Ethical considerations*

Ethical approval to conduct this study was obtained from the University of Sharjah Research Ethics Committee. Participation in this study was voluntary and verbal consent was obtained from all students who agreed to participate. Participants received an information sheet and a full explanation of the purpose of the study. They were assured that their participation would be anonymous and identifying information would not be collected. All data collected were kept confidential and only used for research purposes.

## Results

*Participants’ characteristics*

In total, 241 female students participated in this study; 93 (38.6%) from the Medical campus, 90 (37.3%) from the Women’s campus, and 58 (24.1%) from the Fine Arts and Design campus. Most participants were aged 18–23 years (95.4%) and were single (95.4%). The majority of participants were non-UAE nationals (79.7%), and 29.9% were in their second year of study (Table 1).

*Knowledge about breast cancer*

**Figure 1:**
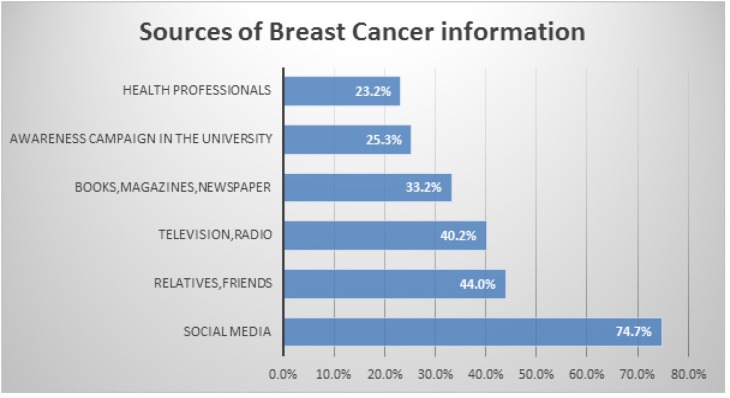
Sources of Information about Breast Cancer

Most (99.2%) participants had heard of breast cancer. The main source of information about breast cancer was social media (74.7%), such as Facebook, Twitter, and Instagram (Figure 1). A family history of breast cancer was reported by 21.6% of participants, and three participants reported a personal history of breast cancer.

*Knowledge about risk factors and warning signs and symptoms*

**Figure 2:**
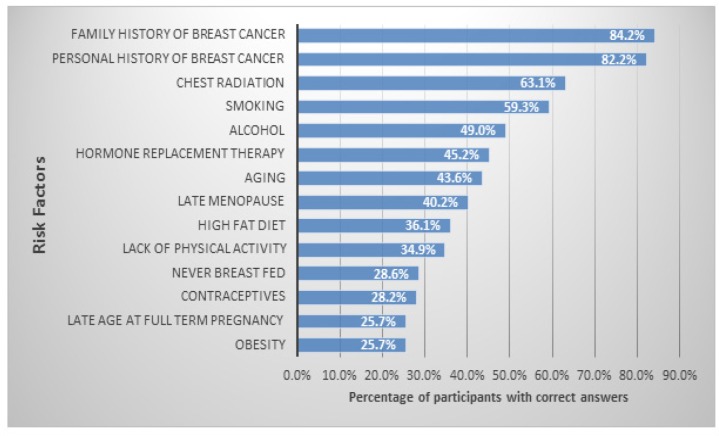
Percentage of Correct Answers Regarding Risk Factors for Breast Cancer

Around half of the participants (49.8%) were knowledgeable about breast cancer risk factors. The risk factors most commonly identified correctly were a family history of breast cancer (84.2%), followed by personal history (82.2%). Chest radiation and smoking were also correctly identified as risk factors by more than half of the participants (63.1% and 59.3%, respectively). Obesity and advanced age at full term pregnancy were the two least-identified risk factors (25.7% for both) (Figure 2).

**Figure 3:**
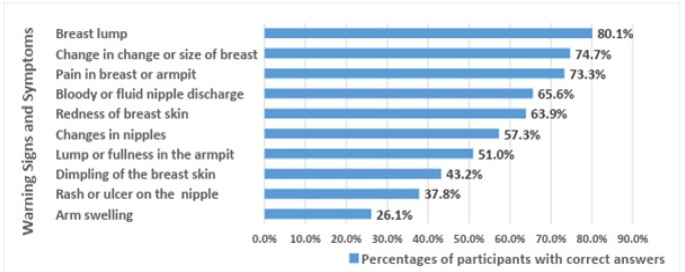
Percentage of Correct Answers Regarding the Warning Signs and Symptoms of Breast Cancer

The majority of participants were non- knowledgeable about the warning signs and symptoms of breast cancer (61.8%), and only 38.2% had adequate knowledge. The warning signs and symptoms most commonly identified correctly were breast lump (80.1%), change in breast size and shape (74.7%), and pain in the breast or armpit (73.3%). Rash or ulcer on the nipple and arm swelling were the least-identified breast cancer warning signs and symptoms (37.8% and 26.1%, respectively) (Figure 3).

There was a significant association between knowledge about risk factors and campus (P=0.000). More participants attending the Medical campus were knowledgeable about breast cancer risk factors (55.0%), followed by the Women’s campus (30.0%) (Table 2). Interestingly, there was no significant association between knowledge about breast cancer warning signs and symptoms and campus (P=0.471). However, more participants at the Women’s (32.6%) and Medical (42.4%) campuses had knowledge about warning signs and symptoms than Fine Arts and Design campus participants (25.0%) (Table 2).

*Knowledge and practice of BSE*

**Figure 4:**
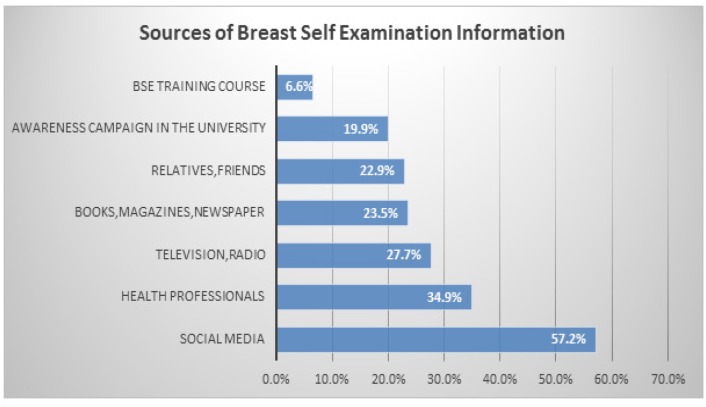
Sources of Information about Breast Self-Examination; BSE, Breast Self-examination

In total, 166 (68.9%) participants had heard of BSE. The Medical campus had the highest percentage of participants who had heard about BSE (45.2%), followed by the Women’s (32.5%) and Fine Arts and Design (22.3%) campuses (Table 2). The main source of information about BSE was social media (57.2%), followed by health professionals (34.9%); however, only 6.6% of participants had undergone a BSE training course (Figure 4). The highest percentage of participants who performed BSE were from the Medical campus (39.1%), with the Women’s and Fine Arts and Design campuses having an equal percentage of participants who performed BSE (30.4%) (Table 2).

Only 62 participants (37.3%) had a correct understanding of what BSE involved; 58.4% had correct knowledge regarding the recommended frequency of performing BSE and 42.8% knew about the right timing for performing BSE (Table 3). Most participants (72.3%) indicated that they did not perform BSE. Common reasons for not performing BSE were “not knowing how” (32.4%), “forgetting” (28.8%), and “not interested” (20.7%). Among participants who performed BSE, most indicated they performed BSE rarely (41.3%), and only 19.6% performed BSE once a month as recommended. In addition, among these participants, only 8.7% were highly confident that they would be able to detect changes in their breasts, with a majority (32.6%) being only slightly confident of detecting any changes (Table 3).

Our results indicated that overall, 213 (88.4%) participants agreed that awareness of breast cancer and BSE should be increased. We found that 38% of participants thought that providing free training courses at the University would be the best method to increase awareness of breast cancer and BSE. In addition, 34% thought that more awareness campaigns would be a good method to increase awareness, and 28% indicated mandatory educational courses regarding breast cancer and BSE would be desirable.

## Discussion

Breast cancer is the most common type of cancer among women in the UAE. Women in the UAE are likely to develop this disease a decade earlier than women in Western countries. Breast cancer awareness and regular practice of BSE facilitate early detection of breast cancer, which improves the chances of survival and better health outcomes. Few studies have investigated knowledge about breast cancer and BSE among female university students in the UAE, and this is the first study conducted among female students at the University of Sharjah. Our study provides useful insights to help address this knowledge gap.

**Table 1:** Participants’ Demographic Characteristics

Demographic characteristics	n (%)
Nationality	
Non-national	192 (79.7)
Local (UAE national)	40 (16.6)
No response	9 (3.7)
Age, years	
18–23	230 (95.4)
24–29	8 (3.3)
30–35	3 (1.2)
Marital status	
Single	230 (95.4)
Married	8 (3.3)
Divorced	3 (1.2)
Campus	
Women’s	90 (37.3)
Medical	93 (38.6)
Fine Arts and Design	58 (24.1)
Academic year	
First	65 (27.0)
Second	72 (29.9)
Third	52 (21.6)
Fourth	50 (20.7)
Fifth	2 (0.8)

**Table 2:** Awareness of Breast Cancer Risk Factors, Signs/Symptoms, and Breast Self-examination by Campus

	Medical campus	Women’s Campus	Fine Arts & Design campus	Total
No. of participants (N=241)	93	90	58	241
Awareness of breast cancer risk factors^a^, n (%)				
Knowledgeable	66 (55.0)	36 (30.0)	18 (15.0)	120 (100)
Non-knowledgeable	27 (22.3)	54 (44.6)	40 (33.1)	121 (100)
Awareness of breast cancer warning signs and symptoms^b^, n (%)				
Knowledgeable	39 (42.4)	30 (32.6)	23 (25.0)	92 (100)
Non-knowledgeable	54 (36.2)	60 (40.3)	35 (23.5)	149 (100)
Participants that had heard about breast self-examination, n (%)				
Yes	75 (45.2)	54 (32.5)	37 (22.3)	166 (100)
No	18 (24.0)	36 (48.0)	21 (28.0)	75 (100)
Participants who performed breast self-examination (N=166), n (%)				
Yes	18 (39.1)	14 (30.4)	14 (30.4)	46 (100)^a^
No	57 (47.5)	41 (34.2)	22 (18.3)	120 (100)

^a^Actual sum of percentages, 99.9% because of rounding.

**Table 3:** Knowledge and Practice of Breast Self-examination among Participants

Knowledge about breast self-examination (BSE)	n (%)
What do you know about the purpose of BSE?	
BSE is to detect lumps in the breast	11 (6.6)
BSE is using your fingers around your breasts to detect lumps	62 (37.3)
Assessment done by doctors/nurses to check for lumps	9 (5.4)
All of the above	84 (50.6)
How often do you think BSE should be performed?	
Daily	4 (2.4)
Weekly	9 (5.4)
Monthly	97 (58.4)
Yearly	22 (13.3)
I don’t know	34 (20.5)
When do you think is the right time for a woman to perform BSE?	
Before menstruation	16 (9.6)
Middle of menstruation (day 3–5)	11 (6.6)
Any day during menstruation	11 (6.6)
After menstruation	71 (42.8)
Anytime	56 (33.7)
No response	1 (0.6)
Practice of BSE	
Do you perform BSE? (N=166)	
Yes	46 (27.7)
No	120 (72.3)
If no, why not? (N=111)	
Fear of positive finding	10 (9.0)
Forgetting	32 (28.8)
Not sure of its ability to detect breast cancer	10 (9.0)
Don’t know how	36 (32.4)
Not interested	23 (20.7)
How often do you check your breasts? (N=46)	
Rarely	19 (41.3)
At least once every 6 months	12 (26.1)
At least once a month	9 (19.6)
At least once a week	2 (4.3)
Don’t know	4 (8.7)
How confident are you that you would notice a change in your breasts? (N=46)	
Not confident	9 (19.6)
Slightly confident	15 (32.6)
Moderately confident	9 (19.6)
Highly confident	4 (8.7)
Don’t know	9 (19.6)

**Figure 5:**
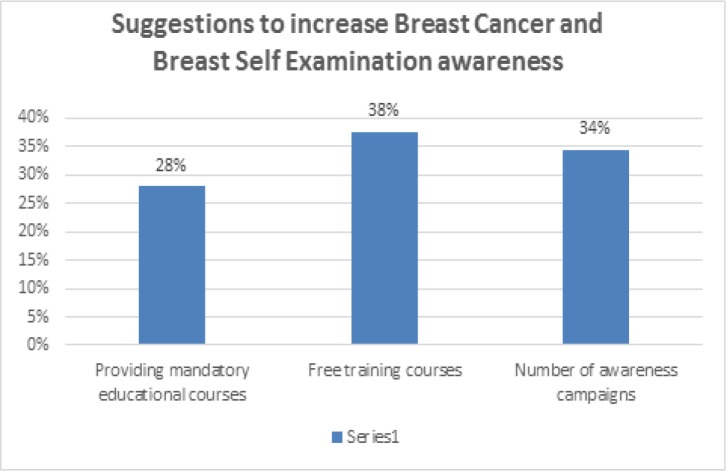
Suggestions to Increase Awareness about Breast Cancer and Breast Self-Examination

We found that most participants (98.8%) had heard of breast cancer, with social media being the most common source of information. This finding was consistent with a study conducted among female students in Egypt that showed mass media (TV and radio) was the main source of information about breast cancer for 89.1% of participants (Boulos and Ghali, 2014). Similarly, a study in Yemen reported mass media was the main source of information for 81.6% of participants (Ahmed, 2010). This may be explained by the similar levels of mobile technology penetration in Egypt and the UAE. Furthermore, our sample was a group of University students, who tend to be technology savvy. 

In our study, around half of the participants were knowledgeable about risk factors and warning signs and symptoms of breast cancer. The most frequently identified risk factor was personal history of breast cancer (82.9%), which was consistent with a study conducted in Turkey among high school students (68.7%) (Karayurt et al., 2008). We also found that students from the Medical campus were more knowledgeable about breast cancer risk factors compared with those from the Women’s and Fine Arts and Design campuses. However, this may be attributable to the topics studied in the Medical campus. This finding was also consistent with a study conducted among medical and non-medical students in Southern Punjab, Pakistan (Sudhanthra and Relton, 2014) that reported medical students’ knowledge about risk factors was significantly better than that of non-medical students, although the overall level of knowledge was insufficient.

Less than half of our participants (40%) were knowledgeable about the warning signs and symptoms of breast cancer. This suggests that the level of knowledge about warning signs and symptoms was insufficient. This was consistent with the study by Sudhanthra and Relton (2014), which reported less than 35% of participants were aware of the early warning signs of breast cancer. Among our participants, “breast lump” was the most commonly identified warning sign/symptom, which was consistent with previous findings (Boulos and Ghali, 2014). In our study, participants from the Women’s campus were more knowledgeable about the warning signs and symptoms of breast cancer than those from the fine Arts and Design campus. However, this finding may be explained by response bias, as the Women’s campus had a higher percentage of participants than the Fine Arts and Design campus. As expected, students from the Medical campus were more knowledgeable than those from the other two campuses. 

Over two-thirds (69.4%) of the participants in this study had heard about BSE, which was similar to the Egyptian study that reported 63.4% of participants had heard about BSE (Boulos and Ghali, 2014). In our study, the highest percentage of participants who had heard about BSE was from the Medical campus, which again may be attributable to their medical background. However, relatively few (40.7%) participants had a correct understanding of what BSE entailed. Similarly, a study conducted among women in Ondo state, Nigeria Makanjuola et al., (2013) reported that only 22% of participants understood what BSE was. We found that almost two-thirds (59.3%) of students that had heard of BSE had correct knowledge regarding the recommended frequency (monthly) and right timing (after menstruation) to perform BSE. However, the study among female university students in Egypt reported that only 8.8% of students knew the appropriate time to perform BSE (Boulos and Ghali, 2014).

A concerning finding in our study was that few (28.8%) participants performed BSE, and most of these participants rarely performed BSE. This finding was consistent with that of a study among female university students in Jordan that reported 11% of participants performed BSE (Suleiman, 2014). The two most common reasons for not performing BSE given by participants in our study were “forgetting” and “do not know how to perform BSE.” The study involving Egyptian students reported similar reasons for not performing BSE, such as “did not know how to perform BSE” and “lack of interest” (Boulos and Ghali, 2014). An encouraging finding from our study was that most students agreed that awareness about breast cancer and BSE should be increased, with popular methods of increasing awareness being free university-based training courses and more overall awareness campaigns.

This study had some limitations that should be considered. We used a purposive sampling method; therefore, our study sample may not be representative of all female students at the University of Sharjah. In addition, our findings cannot be generalized to all female students at the University of Sharjah or more widely to female university students elsewhere, because potential participants did not have a random chance of being selected. Furthermore, the required sample size for this study was calculated at 300 participants, but only 250 participants were approached because of time constraints. 

In conclusion, we found that the level of awareness about breast cancer and BSE varied among female students at the three campuses, with awareness being higher in the Medical campus than in the Women’s and Fine Arts and Design campuses. Although a majority of participants knew about BSE, only 28.81% actually performed the technique. This signals a need for prompt interventions to raise awareness about breast cancer and BSE among female students at the University of Sharjah. Comparisons with similar studies conducted in other countries indicate that the need to raise awareness about breast cancer among female university students is likely to be relevant globally.

Female university students need to be more aware of breast cancer and encouraged to practice BSE regularly to detect abnormalities in their breasts and identify breast cancer at an early stage. Appropriate educational interventions, such as elective courses that cover key aspects of women’s health, may be important for female university students. Providing free BSE training courses may also be an effective way to raise awareness. This study generated new information and insights about the level of awareness about breast cancer and BSE among university students, thereby creating opportunities for further research. It is recommended that further studies conducted in this area use a probability sampling technique to improve the representativeness of the study population and generalizability of the findings, and provide more robust results.

## Funding

No external funding was received for this study.
